# Sleep Supports the Slow Abstraction of Gist from Visual Perceptual Memories

**DOI:** 10.1038/srep42950

**Published:** 2017-02-17

**Authors:** Nicolas D. Lutz, Susanne Diekelmann, Patricia Hinse-Stern, Jan Born, Karsten Rauss

**Affiliations:** 1 Institute of Medical Psychology and Behavioral Neurobiology, University of Tübingen, Otfried-Müller-Straße 25, 72076 Tübingen, Germany; 2 Graduate Training Centre of Neuroscience/IMPRS for Cognitive & Systems Neuroscience, University of Tübingen, Österbergstraße 3, 72074 Tübingen, Germany; 3 Werner Reichardt Centre for Integrative Neuroscience, University of Tübingen, Otfried-Müller-Straße 25, 72076 Tübingen, Germany

## Abstract

Sleep benefits the consolidation of individual episodic memories. In the long run, however, it may be more efficient to retain the abstract gist of single, related memories, which can be generalized to similar instances in the future. While episodic memory is enhanced after one night of sleep, effective gist abstraction is thought to require multiple nights. We tested this hypothesis using a visual Deese-Roediger-McDermott paradigm, examining gist abstraction and episodic-like memory consolidation after 20 min, after 10 hours, as well as after one year of retention. While after 10 hours, sleep enhanced episodic-like memory for single items, it did not affect gist abstraction. One year later, however, we found significant gist knowledge only if subjects had slept immediately after encoding, while there was no residual memory for individual items. These findings indicate that sleep after learning strengthens episodic-like memories in the short term and facilitates long-term gist abstraction.

Sleep benefits consolidation of individual episodes and items in memory^1^. However, memory is not an exact image of past events, but a highly dynamic system that is subject to change and inaccuracies^2^,^3^,^4^. Memories have been conceptualized as flexible constructs that preserve what is most relevant for future needs and that are constantly adapted and updated to fit those needs^2^,^5^. While in the short term, newly encoded information is mostly retained as episodic memories with vivid contextual details, over time those memories become decontextualized and generalized, to form a more abstract representation of the experience^6^. The essence of such an abstract representation has been referred to as a schema or the ‘gist’ of encoded information^7^,^8^. Abstraction of verbal information has been intensely studied using the Deese-Roediger-McDermott (DRM) paradigm^9^,^10^. In this paradigm, subjects learn lists of highly associated words (e.g., white, night, cat, dark etc.), with the ‘gist’ word of the list (in this example: black) not being presented. At later retrieval tests, subjects frequently indicate to ‘remember’ this gist word with high confidence even though they have actually never learned it. With regard to the effect of sleep on gist abstraction in the DRM paradigm, the literature is inconclusive: whereas some studies showed an enhancing effect of sleep on the generation of gist memories^11^,^12^,^13^,^14^, others found no effects or even a reduction of such knowledge after sleep compared to wakefulness^15^,^16^,^17^. Notably, all of these studies examined memory shortly after the critical retention periods of sleep or wakefulness. Assuming that early effects of sleep are expressed in stronger episodic (i.e. veridical) memory, and that gist abstraction is a process that evolves slowly over extended time intervals, the effects of sleep triggering the process of gist abstraction may only become evident after longer delays. While a single night of sleep or even a nap as short as 6 minutes might be sufficient for individual episodic memories to consolidate^18^,^19^, effective integration and abstraction of information within cortical networks might need additional time to evolve^7^,^20^,^21^. Indeed, there are also clues from sleep studies that the decontextualization of memory is a slow and gradual process that takes at least several days, although the role of sleep and its timing during this process is unclear^22^,^23^,^24^,^25^,^26^. Sleep on the night after encoding might be most effective, with improving effects on retrieval still evident after several years^27^. Here, we used a non-verbal, purely visual-perceptual version of the DRM paradigm^28^,^29^,^30^ to examine the effects of sleep during the night after encoding on the evolvement of abstract gist memory. Recall was tested after 20 min, 10 hours (including sleep or wakefulness), as well as after one year of retention. We hypothesized that, whereas sleep leads to an immediate enhancement of veridical item memory, the enhancing effect of post-encoding sleep on gist memory is unmasked only after a longer delay period.

## Results

### 20-min and 10-hour recall

**Figure 1:**
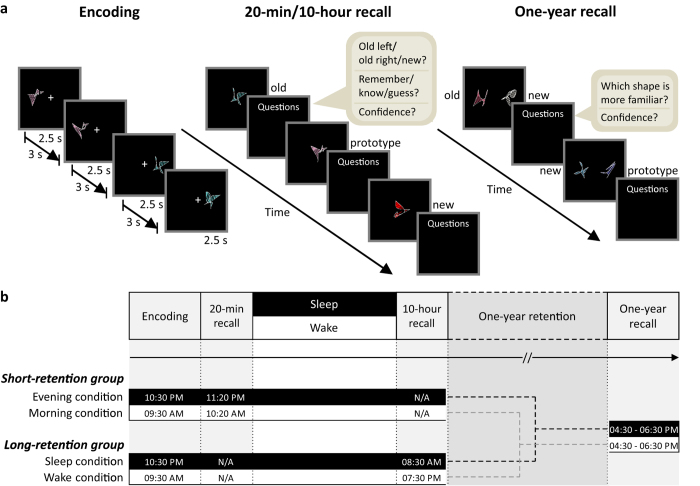
(**a**) Memory task. During encoding, subjects learned 16 item sets, each comprising 10 abstract shapes presented on the left or right side of the screen (only four shapes are shown). At a recall after 20 min or 10 hours, subjects were presented with studied old shapes (item memory), non-studied prototype shapes (gist memory) and new shapes. For each shape they had to indicate whether it was presented during the encoding phase and if so, whether it had been presented on the left or right side. In addition, subjects gave remember/know/guess judgments and rated their confidence on a 4-point scale. At the one-year recall, subjects performed a two-alternative forced-choice task where old shapes versus new shapes or prototypes versus new shapes were simultaneously presented. Subjects had to indicate as quickly as possible which of the two shapes was more familiar to them. After each trial, subjects rated their confidence on a 4-point scale. (**b**) Experimental design. Two groups of subjects were tested in two conditions each, with conditions separated by at least two weeks. In the *Short-retention group*, recall was tested 20 min after encoding, and subjects were tested in counterbalanced order in Morning and Evening conditions. In the *Long-retention group*, recall was tested after 10 hours, and subjects were tested in counterbalanced order in Sleep and Wake conditions. Different sets of stimuli were used for the subjects’ two conditions. A delayed recall test was administered approximately one year after encoding. Here, subjects from the Sleep and Evening conditions and those from the Wake and Morning conditions were pooled for analysis. Shapes were constructed by Scott D. Slotnick, as described in ref. 28.

Twenty-eight subjects were recruited and randomly assigned to either a *Short-retention group* (20 min, *N* = 15) or a *Long-retention group* (10 hours, *N* = 13). All subjects encoded 16 sets of 10 highly associated abstract shapes each, in two retention conditions (i.e. 160 shapes per condition and 320 shapes overall) (Fig. 1a; see also Supplementary Fig. S1 for an example stimulus set). In one condition, the sets were encoded in the morning, and in the other condition, they were encoded in the evening. The order of conditions was randomized. The shapes within each set were derived from a single prototype (the ‘gist’ of the set), which was not shown during encoding. In the *Short-retention group*, memory recall took place 20 min after encoding (Evening and Morning conditions). In the *Long-retention group*, memory recall took place 10 hours after encoding, with this interval including either night-time sleep or daytime wakefulness (Sleep and Wake conditions). The *Short-retention group* thus served as a control for unspecific circadian effects on encoding and/or retrieval (Fig. 1b).

During retrieval, subjects saw stimuli belonging to three different categories: previously encoded old shapes, previously unseen prototype shapes, and completely new shapes. Their task was to indicate for each stimulus if it was old or new. Furthermore, they rated whether their choice reflected explicit remembering, implicit knowing, or random guessing^31^, as well as how confident they were concerning their old/new judgement (4-point scale ranging from 1 (*I had to guess*) to 4 (*absolutely sure*)). The number of prototype shapes categorized as ‘old’ served as a measure of gist memory. The number of correctly recognized old shapes was used to assess item memory. Here, in order to focus on episodic memory aspects, analyses concentrated on those old shapes for which subjects indicated to remember them with high confidence (i.e. ‘remember’ judgments with a confidence rating of 3 or 4), which corresponds to a clear and vivid recollection of the respective item^32^,^33^,^34^,^35^,^36^,^37^ (see Supplementary Table S1 for a comprehensive analysis of item recall). We refer to this measure as ‘high-confidence old shapes’ in the remainder of the manuscript.

**Figure 2:**
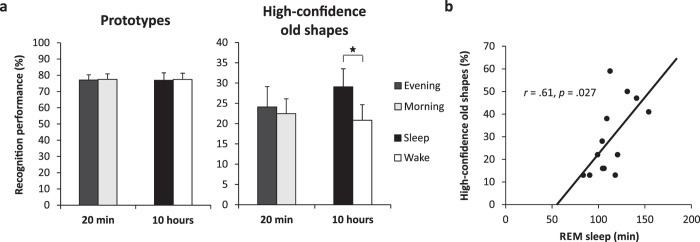
Recall of prototypes (gist memory) and high-confidence old shapes after 20-min (*Short-retention group, N* = 15) and 10-hour (*Long-retention group, N *= 13) retention intervals. (***a***) Memory for prototypes (*left*) did not differ between conditions in both groups. The amount of high-confidence old shapes (*right*) was significantly increased after sleep compared to wakefulness after 10 hours, whereas there was no difference after 20 minutes. Means and SEM are shown. (**b**) Correlation between high-confidence old shapes in the 10-hour Sleep condition and the time (in min) spent in REM sleep. *p < 0.05.

Memory for prototypes, following 20 min or 10 hours of retention, did not differ between conditions, either in the *Short-retention group* (Evening vs. Morning condition, *t*(14) = 0.14, *p* > 0.89), or in the *Long-retention group* (Sleep vs. Wake condition, *t*(12) = −0.08, *p* > 0.93) (Fig. 2a). In contrast, memory for high-confidence old shapes significantly increased after sleep compared to wakefulness in the *Long-retention group (t*(12) = 2.27, *p* = 0.043, *η*^2^ = 0.30) (Fig. 2a). There was no difference between morning and evening recall of high-confidence old shapes in the *Short-retention group (t*(14) = −0.76, *p* > 0.4). Correlation analyses revealed that both time (in min) and percentage (with reference to total sleep time) of REM sleep correlated significantly with high-confidence old shapes (*r* = 0.61, *p* = 0.027 and *r* = 0.57, *p* = 0.043, respectively; Fig. 2b). There were no other significant correlations with any sleep parameters.

Overall analyses of variance, separately conducted for the 20-min and 10-hour groups, showed that both high-confidence old shapes and prototype memory were significantly higher than the rate of ‘old’ responses for new shapes (main effect of Stimulus type in both groups (*F*(2,28) = 58.52, *p* < 0.001 and *F*(2,24) = 46.02, *p* < 0.001, respectively; all *p* < 0.001, for *post-hoc* comparisons; Supplementary Fig. S2). After the 10-hour retention interval, prototype memory rates (as a measure of gist memory) were significantly higher than memory rates of old shapes (as a measure of item memory) (*p* < 0.05), and this difference was not affected by sleep (*p* > 0.7 for both main effect Sleep/wake and interaction Sleep/wake x Stimulus type; see also Supplementary Table S1). In the 20-min group, memory for prototypes and old shapes did not differ (*p* > 0.2). There was no significant difference for any memory measure between the Evening and Morning conditions of the *Short-retention group*, excluding any substantial circadian effects on encoding or retrieval (*p* > 0.8).

### One-year recall

After an average of 386 days, 17 of the original 28 subjects participated in a one-year recall (Fig. 1b). Of these, nine were from the *Short-retention group* and eight from the *Long-retention group*. Their data were combined in the following analyses, as subjects from both groups slept after encoding in one condition (Evening and Sleep conditions, respectively), and stayed awake in the other condition (Morning and Wake conditions, respectively). In other words, the only difference between groups at this delayed recall session is whether initial recall took place before or after a consolidation period of sleep vs. wakefulness. As the latter is a within-subjects factor, this design allows us to efficiently detect effects of immediate vs. delayed sleep on long-term gist abstraction. A potential drawback is that re-activating memories immediately before sleep (i.e. the Evening condition in the *Short-retention group*) might bias gist abstraction. However, the results reported below remained significant even when tested in each group separately.

We used a two-alternative forced-choice task to test for residual memory traces of the encoded material (Fig. 1a). Subjects were simultaneously presented with two stimuli (either an old shape vs. a new shape or a prototype vs. a new shape) and had to indicate as quickly as possible which of the two shapes seemed more familiar.

**Figure 3:**
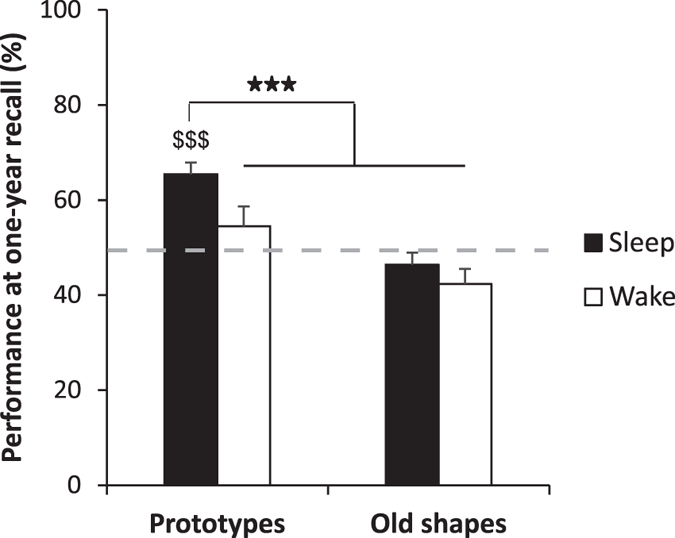
One-year recall. In a two-alternative forced-choice task, above-chance performance was only observed for prototypes when subjects had slept after encoding (^$$$^*p* < 0.001), but not in the Wake condition or for recall of old shapes (item memory). Importantly, subjects showed significantly better memory for prototypes (gist memory) when they had slept after encoding compared to the other three conditions (***p < 0.001). Mean and SEM are shown. *N* = 17.

Separate analyses of memory recall for prototypes and old shapes revealed above-chance performance only for prototype memories after subjects had slept (*t*(16) = 6.25, *p* < 0.001, *η*^2^ = 0.71, Fig. 3). Given that neither recall of old shapes nor recall of prototypes in the Wake/Morning condition reached levels above chance, we used planned contrasts to directly test the hypothesis that sleep specifically supports long-term retention of only the most relevant memories (28), i.e., the prototypes. Indeed, results showed that memory performance for prototypes after sleep was significantly better than in the other three conditions (*t*(16) = 5.75, *p* < 0.001, *η*^2^ = 0.67).

Control analyses confirmed that these differences in recognition performance were independent of the subjects’ original group assignment, as these contrasts were significant in both groups (*t*(8) = 6.25, *p* < 0.001, *η*^2^ = 0.83 and *t*(7) = 3.02, *p* = 0.019, *η*^2^ = 0.57, for *Short-* and *Long-retention group*, respectively). They were further not affected by the length of the individual retention interval between 20-min/10-hour and one-year recall (all *p* > 0.15 for correlations between the length of retention interval and memory of prototypes/old shapes). Pairwise comparisons confirmed that recall of prototype memory was better than recall of memory for old shapes in both sub-conditions forming the Sleep/Evening condition at one-year recall, i.e., for the Sleep condition (*t*(7) = 4.69, *p* = 0.002, *η*^2^ = 0.76) as well as for the Evening condition (*t*(8) = 3.53, *p* = 0.008, *η*^2^ = 0.61). Because subjects saw the prototypes for the first time (at the recall test) in the morning after sleep in the Sleep condition but in the evening before sleep in the Evening condition, these analyses rule out that timing of the first encounter of the prototypes is an essential factor determining the enhancing effect of sleep on long term memory for prototypes. Correlation analyses did not reveal an association of prototype memory after one year with any of the memory measures at the 20-min/10-hour recall (all *r* < 0.33, *p* > 0.19). Similarly, long-term prototype memory did not correlate with either SWS or REM sleep as measured one year before (both |*r*| < 0.34, *p* > 0.41).

In addition to behavioural performance, we recorded eye-movement data during the two-alternative forced-choice task (The Eye Tribe Tracker, The Eye Tribe ApS, Copenhagen, Denmark). A planned contrast testing the hypothesis that this effect was, again, driven by sleep selectively favouring formation of prototype memories, confirmed the behavioural findings (*t*(14) = 3.44, *p* = 0.004, *η*^2^ = 0.46 for the contrast between gaze time for prototypes after sleep compared to the other three conditions, Supplementary Fig. S3). Memory for prototypes after one year was significantly correlated with the percentage of gaze time for prototypes in subjects who slept after encoding (i.e. Sleep/Evening conditions; *r* = 0.57, *p* = 0.025). After post-encoding wakefulness (i.e. Wake/Morning conditions), this correlation was only a trend (*r* = 0.45, *p* = 0.096). No such correlations were evident for recall of old shapes (all *r* ≤ 0.21, *p* > 0.4).

### Sleep parameters and control tests

In the *Long-retention group*, standard polysomnography and assessment of subjective sleep quality were performed in the Sleep condition. Analyses confirmed normal sleep under laboratory conditions (means ± SEM, total sleep time 459.31 ± 1.41 min, sleep stage 1, 5.0 ± 0.19%; stage 2, 53.3 ± 0.47%; slow wave sleep, 15.9 ± 0.37%; REM sleep, 24.7 ± 0.31%).

Before encoding as well as after the 20-min and 10-hour recall, subjects performed a word fluency test^38^ and a working memory test (digit-span task). They were further tested on a vigilance task^39^ and subjective sleepiness (Stanford Sleepiness Scale, SSS^40^) was assessed before and after encoding, before and after the 20-min and 10-hour recall, as well as before the one-year recall. Subjects in the *Short-retention group* showed no significant differences between Evening and Morning conditions for any of the control variables during encoding (all *p* > 0.1) or recall (all *p* > 0.3). Subjects in the *Long-retention group* felt more sleepy in the Sleep condition compared to the Wake condition at encoding (*t*(12) = 2.55, *p* < 0.05). However, vigilance performance (response times) did not differ (*t*(12) = −0.755, *p* > 0.4). In the one-year recall, subjects displayed normal sleepiness and vigilance performance. There were no other differences between retention groups or conditions (see Supplementary Table S2).

## Discussion

We analysed the effects of post-encoding sleep on the temporal evolvement of gist abstraction from individual item memories in a visual DRM paradigm. For this purpose, recall was tested after 20 min, after a 10-hour retention interval including night-time sleep or daytime wakefulness, and again one year later. The number of high-confidence old shapes (old shapes that were remembered with high confidence), reflecting veridical episodic-like memory, was enhanced after the 10-hour interval when sleep followed encoding, compared to wakefulness. This effect of sleep was not seen for prototype memory, reflecting gist abstraction. Conversely, after one year, subjects who had slept after learning showed increased gist memory compared to the Wake condition, whereas there was no evidence of any residual item memory (old shapes) in any of the groups. Eye-movement data obtained at the one-year recall confirmed these findings, showing a higher percentage of gaze time specifically for prototypes in the Sleep condition. In combination, these findings are consistent with the notion of a twofold function of post-encoding sleep: i.e., to strengthen veridical episodic-like memory in the short term, and to support the abstraction of generalized gist knowledge from single episodes in the long term^41^.

According to current concepts of system consolidation^21^, the gist of different experiences is retained for long time intervals, while the memory for single events is forgotten over time. Indeed, in our experiments, gist memory performance was clearly above chance one year after learning only when subjects had slept after encoding, and there was no indication of any significant memory left for single items. This indicates a crucial role of post-learning sleep for retaining gist memories over long time intervals. Such effects of sleep can be considered highly adaptive in order to store a small number of general features of an experience that are relevant for future behaviour, rather than retaining a large number of single cases.

Importantly, even though gist abstraction develops over longer time intervals, the first night of sleep after learning seems to serve a critical function whose behavioural effects are unmasked only later. In our study, the Sleep/Evening and Wake/Morning conditions at the one-year recall solely differed in whether sleep occurred during the first 10 hours after learning, and all groups went into normal sleep-wake cycles for multiple nights thereafter. Thus, the first night after learning might be a critical time window during which sleep can initiate processes of gist abstraction, which then continue to evolve over longer intervals. However, this conclusion is speculative, because our study did not systematically vary sleep during subsequent nights. We also note that gist memory, compared to item memory, was already enhanced after the 10-hour interval, independently of sleep or wakefulness. This pattern is consistent with previous findings^23^ and suggests that abstracting gist is initially a time-dependent process not necessarily requiring sleep. Sleep’s specific role might primarily be to maintain gist for the long term.

Conceptually, the strengthening of episodic memory as well as the abstraction of gist from such representations is thought to originate from the neural reactivation of representations in the hippocampus-dependent declarative memory system^7^,^41^,^42^. This view predicts that strong effects of sleep on episodic memory go along with strong delayed effects on gist abstraction. Contrary to this concept, in the present study gist memory was not correlated with any measure of item memory at the 10-hour recall. However, this lack of correlations does not imply that the two processes are entirely independent of each other. Future studies need to directly address the question whether and in which way such interactions between processes of immediate episodic memory consolidation and processes supporting the long term storage of gist information might be established during post-encoding sleep.

Sleep induced an immediate enhancement, at the 10-hour recall, for high-confidence item memory. Recall of such items reflects hippocampus-dependent episodic memory^43^ known to benefit from sleep^41^,^44^. Retrieval judgements such as remember/know/guess ratings and confidence ratings are assumed to be based on the subject’s conscious access to stored memory traces^45^. Sleep might thus primarily improve accessibility of these traces. Surprisingly, we found enhanced high-confidence item memory to be associated with time spent in REM sleep during the post-encoding night. The importance of slow wave sleep (SWS) for episodic memory consolidation is well established, whereas the function of REM sleep in this process is not clear^44^,^46^. There are a few studies suggesting that REM sleep adds to the effects of SWS on hippocampus-dependent episodic memory^47^,^48^ and also the task modality might matter, i.e., the fact that we tested visual perceptual memories^49^,^50^,^51^,^52^. For instance, naps containing REM sleep have previously been suggested to benefit retention of highly overlapping individual visual perceptual memories by rescuing them from retrograde interference^53^. This issue is clearly in need of further study.

The present findings are unlikely to be caused by circadian influences, although the latter cannot be fully excluded. The *Short-retention group*, which encoded the material in the morning or in the evening and was tested 20 min later, served as a circadian control for the night-sleep and day-wake conditions. There were virtually no differences between the Morning and Evening conditions regarding high-confidence item memory, prototype memory, or any of the control variables (word fluency, digit-span, vigilance and sleepiness). Also, control analyses ruled out that the timing of the first encounter of the prototype stimuli during recall in any way confounded the enhancing effects of initial post-encoding sleep on one-year recall.

Previous studies testing the effects of sleep on gist abstraction in the DRM paradigm have reported mixed results, showing increased, unchanged, or decreased gist memory after a period of post-encoding sleep compared to wakefulness^11^,^12^,^13^,^14^,^15^,^16^,^17^. All of those studies used verbal versions of the DRM and none of those studies tested effects of sleep on recall after a longer delay. Consequently, they do not take into account that the enhancing effect of sleep on gist memory might evolve over longer intervals following the critical sleep period. Against this backdrop, the discrepant findings of the present and previous studies^11^,^12^,^13^,^14^ as to when after the post-learning sleep period the enhancement of gist memory occurs, might be attributed to the different task modalities (non-verbal, visual vs. verbal) in these studies. Also, the outcome with verbal DRM paradigms might be biased by deliberate mnemonic strategies used during encoding^54^,^55^,^56^ to a greater extent than with a purely visual version of the paradigm, as used here^28^,^29^.

Altogether, our results in combination with previous studies using the DRM paradigm suggest the following scenario: Episodic encoding triggers processes of gradual gist abstraction, which initially are solely time-dependent, i.e., equally effective during sleep and wakefulness. However, only when sleep occurs soon after encoding, this process leads to the formation of enduring long-term memory for gist information, with concurrent forgetting of episodic detail. By contrast, post-encoding wakefulness leads in parallel to forgetting of both gist and episodic detail. The central unsolved question arising from this scenario is to determine the factors that govern when the enhancing effect of sleep on gist memory becomes behaviourally manifest.

## Methods

### Participants

Thirty-two healthy adults with regular sleep-wake cycles participated in the experiments. The group size of *N* = 16 in our within-subjects design was determined by a statistical power calculation based on an expected large effect size of *d* = 0.8 (1 – β = 0.8, α = 0.05). They did not take any medication, did not report any neurological or psychological disorders, and had normal or corrected-to-normal visual acuity. Subjects were not allowed to ingest caffeine or alcohol during the days of the experimental sessions and the preceding two days. Three subjects from the *Long-retention group* were excluded from analysis because of a sleep efficiency ≤85% during the experimental sleep night. One more subject from the *Short-retention group* was excluded due to her being an outlier on several different measures (more than two standard deviations from the group mean). Thus, 28 subjects were included in the final analyses (13 in the *Long-retention group* and 15 in the *Short-retention group*). In the Sleep condition of the *Long-retention group*, subjects spent an adaptation night in the sleep laboratory, including placement of electrodes. All subjects gave written informed consent and were paid for participation. The experiment was approved by the ethics committee of the Medical Faculty at the University of Tübingen and conducted in accordance with the approved guidelines.

### Design and procedure

Two groups of subjects participated in the experiment, with a delay between encoding and memory recall of 20 min (*Short-retention group*) or 10 hours (*Long-retention group*), and a delayed recall after about one year of retention (both groups). In both groups, subjects were tested in two experimental sessions, separated by at least two weeks, in a within-subjects design: the Evening and Morning condition for the *Short-retention group*, and the Sleep and Wake condition for the *Long-retention group* (Fig. 1b).

In the Sleep/Evening conditions, subjects arrived at the laboratory at 0900 PM. After placement of electrodes for sleep recordings (*Long-retention group* only), they were given several control tasks and questionnaires, before they performed the experimental task. The encoding phase lasted about 30 min from 1030 PM to 1100 PM. After encoding, subjects in the *Short-retention group* listened to relaxing music for 20 min, followed by the 20-min recall and another set of control tests. They then left the laboratory for a normal night of sleep at home. Subjects in the *Long-retention group* were allowed to sleep for eight hours after encoding in the sleep laboratory (until 0700 AM), monitored by standard polysomnography. The next morning, they were allowed to take a shower and had a standardized breakfast, before the 10-hour recall took place at 0830 AM (i.e. 10 hours after encoding). After the recall session, subjects performed another set of control tests.

In the Wake/Morning conditions, subjects arrived at the laboratory at 0900 AM in the morning, performed the control tasks, and filled out the questionnaires. The encoding phase took place between 0930 AM and 1000 AM. After encoding, subjects in the *Short-retention group* listened to relaxing music for 20 min, followed by the 20-min recall and another set of control tests. They then left the laboratory for a normal day of wakefulness. Likewise, subjects in the *Long-retention group* left the laboratory after encoding to engage in their everyday activities. They were instructed not to take any daytime naps during the retention interval, which was controlled by actimetry (Actiwatch^®^ system Cambridge Neurotechnology, Cambridge, UK). Additionally, their daily activities were recorded via questionnaire. Subjects in the *Long-retention group* returned to the laboratory at 0715 PM in the evening and 10-hour recall took place at 0730 PM, before they performed another set of control tests. Since times of encoding in the *Short-retention group* were equal to times of encoding in the *Long-retention group,* the *Short-retention group* additionally serves as a control for circadian effects on initial encoding for the *Long-retention group*.

Seventeen subjects from the original sample (nine from the *Short-retention group* and eight from the *Long-retention group*) participated in a delayed recall test about one year after encoding (mean: 386 days, range: 178 to 542 days). All subjects were tested between 0430 PM and 0630 PM. In this one-year recall test, we also tracked subjects’ eye movements using an Eye Tribe Tracker (The Eye Tribe ApS, Copenhagen, Denmark). Eye-movement data from two subjects could not be analysed due to very low calibration quality (20% and 40%, respectively).

### Memory task

To induce the generation of gist memories, we used a nonverbal version of the DRM paradigm^9^,^10^ introduced by Slotnick & Schacter (2004)^28^ (see also Diekelmann *et al*.^29^). During encoding, subjects observed 16 distinct sets of 10 highly associated abstract shapes each (Fig. 1a). Shapes from the same set had similar outlines and were filled with lines of the same colour and orientation (see Supplementary Fig. S1 for an example stimulus set). Stimuli from each set were presented consecutively on a black background for 2.5 s each, with an inter-trial interval (ITI) of 3 s. All stimuli from one set were consecutively presented either on the left or on the right side of the screen, with the location of each set counterbalanced across individuals. Subjects were instructed to memorize both the shapes and their spatial location. The 10 shapes of each set were derived from a single prototype (the ‘gist’ of each set) which was not shown during encoding. For the two experimental conditions, two parallel versions of 16 different sets of shapes were constructed and the two versions were counterbalanced across conditions.

During 20-min and 10-hour recall, subjects were presented with three types of shapes: 32 studied exemplars (i.e. two old shapes from each of the 16 studied sets), 32 nonstudied exemplars (i.e. new shapes from nonstudied sets), and 16 prototypes (i.e. one prototype shape for each studied set). The shapes were presented in a pseudo-randomized order in the centre of the screen, and subjects were asked to indicate for each shape whether it had been presented during the encoding phase, and if so, whether it had been located on the left or right side of the screen (i.e. response alternatives were ‘old left,’ ‘old right,’ and ‘new’). As there was no effect of position recall between groups and conditions (overall mean ± SEM: 0.71 ± 0.02), data were collapsed across this measure. For each response, subjects additionally rated their confidence on a 4-point scale ranging from 1 (*I had to guess*) to 4 (*absolutely sure*) and gave remember/know/guess judgments for the shapes they identified as ‘old’^31^.

At one-year recall, subjects performed a two-alternative forced-choice task, in which they were simultaneously presented with two stimuli (old shapes vs. new shapes and prototype shapes vs. new shapes) (Fig. 1a). Their task was to indicate which of the two shapes was more familiar to them. After each trial, subjects additionally rated their confidence on a 4-point scale. As we expected overall low confidence ratings, no additional remember/know/guess judgements were collected. Set combinations that were originally presented during encoding in the two different conditions (i.e. the two different versions of shape sets) were presented sequentially, i.e. subjects first saw the stimulus sets of one condition (e.g. Wake), followed by the stimulus sets of the other condition (e.g. Sleep), with the order of stimulus sets counterbalanced across subjects and conditions. Furthermore, the old shapes that were used for the two-alternative forced-choice task were different from those presented during 20-min/10-hour recall, such that subjects saw both old and prototype shapes an equal number of times. Moreover, the number of old shapes matched the number of prototype shapes (i.e. 16 old shapes and 16 prototypes per condition). The shapes’ position on the screen (left or right side) was balanced for both old and prototype shapes. New shapes were completely new, i.e. they had not been presented during 20-min/10-hour recall, and different new shapes were presented on each trial. During both 20-min/10-hour recall and one-year recall, subjects were instructed to respond as quickly as possible without sacrificing accuracy. There was no time limit for responses. Stimulus presentation was performed using Presentation^®^ (Neurobehavioral Systems, Inc., Berkeley, CA, USA).

### Polysomnographic recordings and sleep scoring

Polysomnographic recordings were obtained and digitized at a sampling rate of 200 Hz using a BrainAmp MR plus system (Brain Products, Germany). Electroencephalography (EEG) was recorded at two locations (C3 and C4, according to the international 10–20 system), referenced online against the mean of the two mastoids (A1, A2). Additionally, the electrooculogram (EOG) and electromyogram (EMG, electrodes positioned on the chin) were recorded with bipolar montages. All data were stored unfiltered. For analysis, filters were applied to the EEG and EOG at a frequency of 0.16 Hz (high-pass) and 30 Hz (low-pass) as well as to the EMG at a frequency of 5.31 Hz (high-pass) and 90 Hz (low-pass). In addition, a notch filter was applied at 50 Hz. Sleep scoring was performed offline and manually based on 30-seconds epochs, in accordance with standard criteria^57^.

### Statistical analysis

Memory performance was analysed based on percentages of ‘old’ responses to prototypes (as a measure of gist memory), old shapes (as a measure of item memory), and new shapes. To focus on episodic memory aspects, we also examined the subset of correct item memories for which subjects indicated ‘remembering’ (on the remember/know/guess scale^31^) the specific item with high confidence (3 or 4 on the 4-point confidence scale). To examine the effect of sleep vs. wakefulness and evening vs. morning encoding on gist abstraction and item memory consolidation in the 20-min/10-hour recall, we performed two 3 × 2 repeated-measures analyses of variance (ANOVA) with the factors Stimulus type (prototypes vs. old shapes vs. new shapes) and Condition (Evening vs. Morning for the *Short-retention group*, and Sleep vs. Wake for the *Long-retention group*). To assess relationships between gist memory and item memory as well as relationships between sleep stages and memory performance, Pearson’s *r* was calculated. Given their exploratory nature, no correction for multiple comparisons was performed on the correlations between behavioural performance and sleep stages.

For the one-year recall, data from the *Short-retention group* and the *Long-retention group* were pooled as both groups slept after encoding in one condition (Sleep/Evening conditions) and stayed awake in the other condition (Wake/Morning conditions). Testing against chance level was performed using one-sample *t*-tests. Planned contrasts were used to test the effect of sleep on memory for prototypes against the remaining conditions (prototypes/wake, old shapes/sleep, and old shapes/wake) at the one-year recall, for both analysis of behavioural and eye tracker data. To examine the influence of the delay between 20-min/10-hour recall and one-year recall on performance, we calculated correlations between the delay (number of days) and memory performance. For the eye tracker analysis, regions of interest (ROIs) were defined as the areas covering the shapes on the left and right side of the screen, respectively, and allocated to the different stimulus types. The percentage of gaze time was then calculated as the overall time of fixations falling on either ROI, divided by the total time of stimulus presentation per trial. For ANOVAs, Greenhouse-Geisser correction of degrees of freedom was applied where appropriate. All *post-hoc* tests and further analyses used *t*-tests. Two-tailed tests were chosen for all statistical analyses, unless noted otherwise. The level of significance was set to *p* = 0.05.

### Data availability

The datasets analysed during the current study are available from the corresponding author on reasonable request.

## Additional Information

**How to cite this article:** Lutz, N. D. *et al*. Sleep Supports the Slow Abstraction of Gist from Visual Perceptual Memories. *Sci. Rep.* **7**, 42950; doi: 10.1038/srep42950 (2017).

**Publisher's note:** Springer Nature remains neutral with regard to jurisdictional claims in published maps and institutional affiliations.

## Supplementary Material

Supplementary Information
