# Supplementary material for: Sleep Supports the Slow Abstraction of Gist from Visual Perceptual Memories
Source: Sci Rep. 2017 Feb 17;7:42950. doi: 10.1038/srep42950 (PMC5314355; doi:10.1038/srep42950)
Supplement: Supplementary Information [file srep42950-s1.pdf]

Supplementary Information

Sleep Supports the Slow Abstraction of Gist from Visual Perceptual Memories

Nicolas D. Lutz[1,2], Susanne Diekelmann[1], Patricia Hinse-Stern[1], Jan Born[1,3], and Karsten Rauss[1,*]

[1]Institute of Medical Psychology and Behavioral Neurobiology, University of Tübingen, Otfried-Müller-Straße 25, 72076 Tübingen, Germany, [2]Graduate Training Centre of Neuroscience / IMPRS for Cognitive & Systems Neuroscience, University of Tübingen, Österbergstraße 3, 72074 Tübingen, Germany, [3]Werner Reichardt Centre for Integrative Neuroscience, University of Tübingen, Otfried-Müller-Straße 25, 72076 Tübingen, Germany

* Correspondence and requests for materials should be addressed to K.R. (email: karsten.rauss@uni-tuebingen.de).

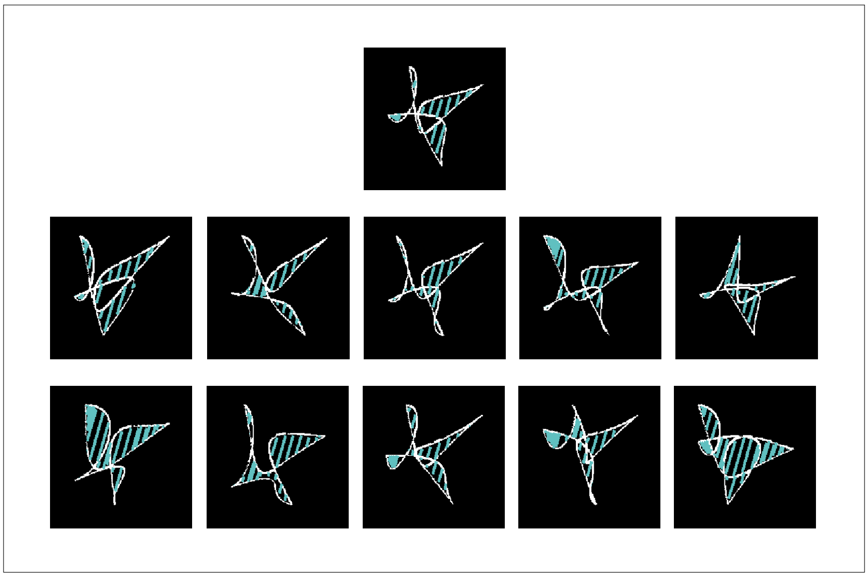

Supplementary Fig. S1. Example stimulus material. The figure shows one set comprising the prototype (*top row*) and the 10 individual items (*bottom rows*). In a pilot experiment, five subjects rated the prototypes (out of three choices) to have most overlap with all other shapes of each set (significant above-chance level effect, mean correct responses: 60.0 ± 5.2%, chance level: 33.3%; $t(4) = 11.54$, $p < .001$).

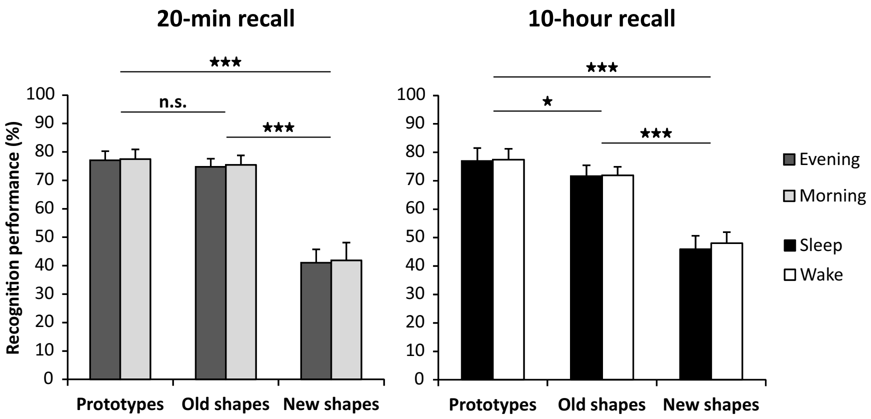

Supplementary Fig. S2. Recognition performance at 20-min and 10-hour recall of non-studied prototype shapes (gist memory), studied old shapes (item memory) and non-studied new shapes in the *Short-retention group* (20 min) and the *Long-retention group* (10 hours). After 20 min (*left*), both memory recall for prototypes and old shapes were significantly higher than the rate of new shapes, but the difference between the rate of prototypes and old shapes was not significant. After 10 hours (*right*), both memory of prototypes and old shapes were significantly higher than the rate of new shapes and prototype memory was significantly higher than memory of old shapes. Mean and SEM are shown. *$p < .05$; ***$p < .001$; n.s., not significant. $N = 15$ and 13 for *Short-retention group* and *Long-retention group*, respectively.

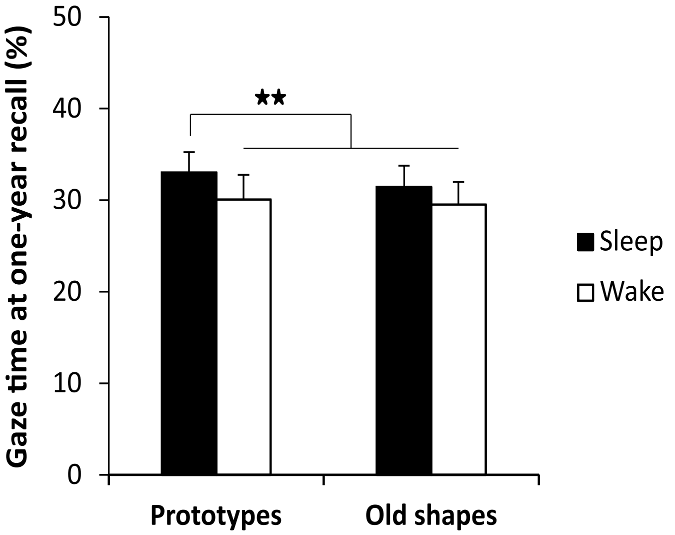

Supplementary Fig. S3. Eye tracker results on the delayed two-alternative forced-choice task after one year of retention. The figure shows the percentage of gaze time for the respective target stimuli on the screen (i.e. prototypes or old shapes as compared to new shapes). Subjects showed a higher percentage of gaze at the prototypes when they had slept after encoding compared to the other three conditions. This difference was due to a significant difference between sleep and wakefulness for prototypes, but not for old shapes. Mean and SEM are shown. $^{**}p < .01$. $N = 15$.

Supplementary Table S1. Recognition memory performance, confidence ratings and remember/know/guess judgements in the 20-min and 10-hour recall.

		Short-retention group (20 min)		*Long-retention group* (10 hours)	
		Evening	Morning	Sleep	Wake
Recognition	Prototypes	0.77 ± 0.03	0.78 ± 0.03	0.77 ± 0.05	0.77 ± 0.04
	Old shapes	0.75 ± 0.03	0.75 ± 0.03	0.72 ± 0.04	0.72 ± 0.03
	New shapes	0.41 ± 0.05	0.42 ± 0.06	0.46 ± 0.05	0.48 ± 0.04
Confidence	Prototypes	2.73 ± 0.10	2.72 ± 0.11	2.94 ± 0.12	2.79 ± 0.15
	Old shapes	2.65 ± 0.12	2.65 ± 0.09	**2.79 ± 0.13**	**2.59 ± 0.15***
	New shapes	**2.17 ± 0.08**	**2.33 ± 0.10***	2.38 ± 0.12	2.36 ± 0.12
Remember	Prototypes	0.39 ± 0.08	0.31 ± 0.07	0.55 ± 0.07	0.48 ± 0.08
	Old shapes	0.35 ± 0.07	0.34 ± 0.06	**0.50 ± 0.06**	**0.41 ± 0.06$^{(*)}$**
	New shapes	0.17 ± 0.04	0.15 ± 0.06	0.27 ± 0.06	0.27 ± 0.05
Know	Prototypes	0.35 ± 0.07	0.46 ± 0.06	0.35 ± 0.06	0.35 ± 0.06
	Old shapes	0.35 ± 0.05	0.38 ± 0.05	0.40 ± 0.06	0.41 ± 0.05
	New shapes	0.37 ± 0.06	0.39 ± 0.08	0.45 ± 0.06	0.41 ± 0.07
Guess	Prototypes	0.26 ± 0.08	0.23 ± 0.05	0.10 ± 0.02	0.17 ± 0.04
	Old shapes	0.30 ± 0.08	0.28 ± 0.06	0.10 ± 0.03	0.18 ± 0.04
	New shapes	0.46 ± 0.08	0.46 ± 0.09	0.28 ± 0.05	0.32 ± 0.07

Asterisks indicate significant or marginally significant differences for comparisons between Evening and Morning conditions or Sleep and Wake conditions, respectively ($^{*}p < .05$; $^{(*)}p < .1$). Mean ± SEM are shown. $N = 15$ and 13 for *Short-retention group* and *Long-retention group*, respectively. As there was no effect of position recall (i.e. whether shapes were presented on the left or right side of the screen) between groups and conditions (overall correct responses: mean ± SEM: 0.71 ± 0.02), data were collapsed for this measure.

Supplementary Table S2. Control variables during encoding and recall.

		Short-retention group (20 min)		*Long-retention group* (10 hours)	
		Evening	Morning	Sleep	Wake
	Age (years)	23.67 ± 0.89		23.38 ± 0.76	
Encoding	Word Fluency (# of words)	20.40 ± 1.33	18.60 ± 0.84	20.69 ± 1.72	19.31 ± 1.52
	Digit Span	7.53 ± 0.41	7.20 ± 0.22	6.23 ± 0.28	6.38 ± 0.40
	Vigilance (mean RT in ms)	416.13 ± 10.15	416.20 ± 12.38	374.54 ± 12.30	382.11 ± 12.02
	Sleepiness (SSS)	2.80 ± 0.28	2.70 ± 0.23	**2.88 ± 0.22**	**2.58 ± 0.20***
20-min/ 10-hour recall	Word Fluency (# of words)	20.67 ± 1.23	19.67 ± 0.98	19.85 ± 1.53	20.92 ± 1.27
	Digit Span	6.87 ± 0.19	6.93 ± 0.25	6.62 ± 0.27	6.92 ± 0.31
	Vigilance (mean RT in ms)	428.87 ± 9.62	428.18 ± 13.87	384.53 ± 14.40	377.98 ± 11.53
	Sleepiness (SSS)	2.87 ± 0.26	2.73 ± 0.18	2.23 ± 0.19	2.15 ± 0.28
One-year recall	Vigilance (mean RT in ms)	436.80 ± 7.37			
	Sleepiness (SSS)	2.53 ± 0.23			

Asterisks indicate significant differences for comparisons between Evening and Morning conditions or Sleep and Wake conditions, respectively (*$p < .05$). Mean ± SEM are shown. RT, reaction time; SSS, Stanford Sleepiness Scale. Values for the SSS are averaged across the two tests before and after encoding as well as before and after 20-min/10-hour recall, respectively. At one-year recall, vigilance and sleepiness data were obtained only once for both conditions. $N = 15$ and 13 for *Short-retention group* and *Long-retention group* in the 20-min/10-hour recall, respectively. $N = 17$ in the one-year recall.
